# “They Told Me to Come Back”: Women’s Antenatal Care Booking Experience in Inner-City Johannesburg

**DOI:** 10.1007/s10995-012-1019-6

**Published:** 2012-04-19

**Authors:** Ijeoma Solarin, Vivian Black

**Affiliations:** 1 Wits Reproductive Health and HIV Institute, University of the Witwatersrand, Johannesburg, South Africa; 2 Wits Reproductive Health and HIV Institute, Hillbrow Health Precinct, Hugh Solomon Building, PO Box 18512, Hillbrow, 2038 South Africa

**Keywords:** Antenatal care, South Africa, Late ANC access, ANC booking experience, PMTCT, HIV

## Abstract

To assess women’s experience of public antenatal care (ANC) services and reasons for late antenatal care attendance in inner-city Johannesburg, South Africa. This cross-sectional study was conducted at three public labour wards in Johannesburg. Interviews were conducted with 208 women who had a live-birth in October 2009. Women were interviewed in the labour wards post-delivery about their ANC experience. Gestational age at first clinic visit was compared to gestational age at booking (ANC service provided). ANC attendance was high (97.0 %) with 46.0 % seeking care before 20 weeks gestation (early). Among the 198 women who sought care, 19.2 % were asked to return more than a month later, resulting in a 3-month delay in being booked into the clinic for these women. Additionally 49.0 % of women reported no antenatal screening being conducted when they first sought care at the clinic. Delay in recognizing pregnancy (21.7 %) and lack of time (20.8 %) were among the reasons women gave for late attendance. Clinic booking procedures and delays in diagnosing pregnancy are important factors causing women to access antenatal care late. In a country where a third of pregnant women are HIV infected, early ANC is vital in order to optimise ART initiation and thereby reduce maternal mortality and paediatric HIV infection. It is therefore imperative that existing antenatal care policies are implemented and reinforced and that women are empowered to demand better services.

## Introduction

The maternal mortality ratio (MMR) and infant mortality rate (IMR) in South Africa are high at 410 per 100,000 live births and 56 per 1,000 live births respectively [1]. These figures stand in contrast to most developed countries where MMR is around 10 per 100,000 births or less and IMR about 5 per 1000 births. Both South Africa’s MMR and IMR are almost five times greater than the average found among countries with similar income levels [2]. There are several contributing factors to both infant and maternal mortality and morbidity in South Africa but the contribution made by HIV is significant. According to the *National Committee on Confidential Enquiries into Maternal Deaths* [3], AIDS is the leading cause of maternal mortality in South Africa, overtaking other direct obstetric causes. Likewise, infant mortality due to HIV is high at an estimated 57.1 %, compared to less than 0.5 % in most developed countries [1].

While the exact content of antenatal care (ANC) and its ability to reduce maternal mortality may not be fully established, there is consensus that quality ANC has an important role to play in maternal and infant health and survival [4, 5]. South Africa’s *Saving Babies* report lists non-attendance and late attendance for ANC as among the top five avoidable causes of perinatal deaths [6] while the *Saving Mothers* report identifies non-attendance and infrequent attendance for ANC as two of the common causes of patient related maternal mortality [3].

Both the South African department of health (DOH) and the World Health Organisation (WHO) recommend women access ANC as soon after realising they are pregnant and definitely before 5 months (20 weeks) gestation [7, 8]. Early attendance for ANC facilitates prompt identification of high risk pregnancies and other potential maternal or foetal problems and allows for early interventions to be initiated.

One such important intervention is the early initiation of antiretroviral therapy (ART) for HIV-positive mothers [9]. This is particularly important for South Africa where antenatal HIV-sero-prevalence is 30.2 % [10]. In most developed countries ART has been shown to decrease mother-to-child transmission (MTCT) rates to below one percent [11], however, studies in South Africa have shown that HIV-transmission rates, while greatly reduced, are still sub-optimal at about five to seven percent [12, 13]. These studies showed that shorter duration of ART during pregnancy was a significant predictor for transmission. They propose that one of the reasons for the inadequate duration of ART is late access to care.

Although South Africa has demonstrably high levels of ANC coverage, late start of antenatal care remains a problem and indeed has been documented in several studies from around the continent [14–18]. Despite this, there is still a lack of information about the ANC experiences of women who access public ANC services in Johannesburg. This study seeks to gain a better understanding of the experiences of these women and assess reasons for late access to ANC.

## Methods

A descriptive cross-sectional study was conducted involving women 18 years and over who had a live birth at one of three public healthcare facilities serving inner-city Johannesburg: one tertiary hospital, one secondary hospital and one primary care centre. These are the only three government facilities in the area with labour wards and are serviced by approximately 20 primary health care clinics in the surrounding metropolitan area with an estimated population of 800,000. Women delivering at these facilities tend to come from lower socio-economic backgrounds.

Antenatal care is free in South Africa and current guidelines state that all primary healthcare facilities should screen pregnant women at their first presentation to the clinic, ideally before 20 weeks gestation. On diagnosing/suspecting pregnancy, women are meant to seek care at their designated local antenatal clinic as soon as possible for same day service. This service should be available Monday to Friday from 0700 h to 1600 h. Nevertheless, anecdotal evidence suggests women may not receive screening and care at their first contact with the clinic. With this in mind, we defined first clinic visit as the first time a woman attended an antenatal clinic seeking ANC and booking visit as the visit in which any antenatal screening was conducted.

Interviews were conducted on 208 women in October 2009. The number of participants recruited from the three centres was proportionally representative to the size of the site based on the annual number of births at each site. Recruitment was conducted from the labour wards in order to identify women who had not attended antenatal care. Furthermore, having completed their pregnancy, it was considered that women would be able to give a more complete account of the care they received with less concern about their care being affected. Women were interviewed in the ward post-delivery and individually while waiting to be discharged.

### Data Collection

Two interviewers with knowledge of several South African languages conducted the interviews. A pre-tested questionnaire was verbally administered to each individual in English or was translated by the interviewers into the participant’s preferred language. Most interviewees who were uncomfortable being interviewed in English spoke Zulu or Xhosa. The questionnaire included sections on demographics, attendance at an ANC clinic, reasons for attendance or non-attendance and experience of care received. Gestational age at first clinic attendance was self-reported and distinctions were made between first clinic visit and booking visit.

### Data Analysis

Data analysis was conducted using STATA 10.0 for windows (Stat Corp, tx USA). As this was a descriptive study, Chi-square tests were conducted on categorical variables with Fisher’s exact test employed where appropriate.

For the purpose of this study and in accordance with DOH recommendations, women who reported attending an antenatal clinic for their first clinic visit at or before they were 5 months (20 weeks) pregnant were categorised as attending early while those who attended after they were 5 months were categorised as late.

The study was approved by the Human Research Ethics Committee of the University of the Witwatersrand (M090817), study site facility managers and by the Gauteng Province. All participants were consented prior to being interviewed.

## Results

### Socio Demographic Characteristics

Mean age was 26.7 years (SD = 5.7). Most women interviewed were unmarried (68.8 %), multigravid (68.8 %), and unemployed (57.7 %) (Table 1). In addition, there was a high level of unplanned pregnancies (56.5 %), and of these, 16.2 % said they were unhappy when they found out they were pregnant. One hundred and thirty-nine women (67.2 %) reported living in their current area for <5 years and 91.8 % would not describe the Johannesburg metropolitan area as their real home indicating that a vast majority of women had relocated from another geographical area.

**Table 1:** Maternal sociodemographic characteristics stratified by attendance (early, late and not attend) and comparing characteristics of women who attended early versus late

Characteristic (N = 208)	Total (%)	Not attend (%)	Early (≤5 months) (%)	Late (>5 months) (%)	*p*-value (early vs. late)
Gravida					χ^2^ (3, 198) = 4.64, *p* = 0.556
1	65 (31.3)	3 (4.6)	34 (52.3)	28 (43.1)	
2	79 (38.0)	4 (5.1)	29 (36.7)	46 (58.2)	
3	46 (22.1)	2 (4.4)	22 (47.8)	22 (47.8)	
≥4	18 (8.7)	1 (5.6)	6 (33.3)	11 (61.1)	
Marital status					χ^2^ (3, 198) = 1.17, *p* = 0.556
Single	70 (33.6)	5 (7.1)	30 (42.9)	35 (50.0)	
Living together	73 (35.1)	3 (4.1)	29 (39.7)	41 (56.2)	
Married	65 (31.3)	2 (3.1)	32 (49.2)	31 (47.7)	
Population group					Fisher’s exact test = 0.756
Black	197 (94.7)	9 (4.6)	87 (44.2)	101 (51.3)	
Other ethnicity	11 (5.3)	1 (9.1)	4 (36.4)	6 (54.5)	
Education					χ^2^ (1, 198) = 5.37, *p* = 0.020*
No school to < secondary school completion	100 (48.1)	4 (4.0)	36 (36.0)	60 (60.0)	
≥Secondary school completion	108 (51.9)	6 (5.6)	55 (50.9)	47 (43.5)	
Work status					Fisher’s exact Test = 0.025*
Paid employee	77 (37.0)	2 (2.6)	28 (36.4)	47 (61.0)	
Schooling/unemployed	120 (57.7)	7 (5.8)	55 (45.8)	58 (48.3)	
Self employed	11 (5.3)	1 (9.1)	8 (72.7)	2 (18.2)	
Yearly income					χ^2^ (3, 198) = 3.71, *p* = 0.295
Don’t know/prefer not to say	33 (15.9)	4 (12.1)	17 (51.5)	12 (36.4)	
<19,200	44 (21.1)	3 (6.8)	16 (36.4)	25 (56.8)	
R19,201- R38, 400	58 (27.9)	1 (1.7)	23 (39.7)	34 (58.6)	
>R38,400	73 (35.1)	2 (2.7)	35 (48.0)	36 (49.3)	
Type of residence*#*					Fisher’s exact test = 0.128
Informal dwelling	8 (3.9)	0 (0.0)	4 (50.00)	4 (50.00)	
Flat in block of flats	115 (55.5)	5 (4.4)	45 (39.1)	65 (56.5)	
House/flat/room in backyard	19 (9.2)	2 (10.5)	6 (31.6)	11 (57.9)	
House/townhouse	65 (31.4)	3 (4.6)	36 (55.4)	26 (40.0)	
Housing situation					χ^2^ (2, 198) = 4.31. *p* = 0.116
Own home	33 (15.9)	1 (3.0)	20 (60.6)	12 (36.4)	
Occupied rent-free	16 (7.7)	1 (6.3)	7 (43.8)	8 (50.0)	
Rent	159 (76.4)	8 (5.0)	64 (40.3)	87 (54.7)	
Length of time in South Africa					χ^2^ (2, 198) = 0.10, *p* = 0.950
≤1 year	13 (6.3)	0 (0.0)	6 (46.1)	7 (53.9)	
>1 year ≥ 5 years	51 (24.5)	3 (5.9)	23 (45.1)	25 (49.0)	
>5 years	144 (69.2)	7 (70.0)	62 (44.5)	75 (55.5)	
Length of time in current area*#*					χ^2^ (2, 197) = 0.60. *p* = 0.742
≤1 year	40 (19.3)	0 (0.0)	20 (50.0)	20 (50.0)	
>1 year ≥ 5 years	99 (47.8)	9 (9.1)	39 (39.4)	51 (51.5)	
>5 years	68 (32.9)	1 (1.5)	32 (47.0)	35 (51.5)	
Country of birth					χ^2^ (1, 198) = 0.01, *p* = 0.928
South Africa	122 (58.7)	6 (4.9)	53 (43.4)	63 (51.6)	
Other	86 (41.3)	4 (4.7)	38 (44.2)	44 (51.2)	
Would describe someplace else as home*#*					χ^2^ (1, 197) = 0.01, *p* = 0.905
No	17 (8.2)	0 (0.0)	8 (47.1)	9 (52.9)	
Yes	190 (91.8)	10 (5.3)	82 (43.2)	98 (51.6)	

*Significant at *p* < 0.05, #1 missing response

### Behaviour Around Pregnancy Discovery

Overall ANC attendance was high, with 95.2 % attending an antenatal clinic at least once and 46.0 % seeking care at or before they were 5 months pregnant. Early attendance (before 5 months) was more often observed among women who had completed secondary education than women who had not (53.9 % vs. 37.5 (χ^2^(1, 198) = 5.37, *p* = 0.02)) and among self-employed women (80.0 %) compared to paid employees (37.3 %) and those describing themselves as unemployed (48.7 %) (Fisher’s exact, *p* = 0.03). In addition women reported being seen a median of five times for care (inter-quartile range (IQR) 3-7) and 73.2 % were seen at least four times during their pregnancy. Among women who attended ANC, a fifth (20.7 %) reported seeking care at more than one ANC clinic.

To determine their pregnancy, 35.4 % of women made use of a self-administered pregnancy test, 30.1 % did not use a test to confirm pregnancy but rather concluded they were pregnant following a few missed periods or relied on other signs of pregnancy such as foetal movement or abdominal swelling or did not realise they were pregnant until they were admitted into hospital, 18.0 % confirmed at a general practitioners (GP) and 16.5 % tested at a government antenatal clinic. When asked about the first action they took following the realisation that they were pregnant, 57.1 % reported that they sought care at an antenatal clinic or primary healthcare facility, 26.0 % visited their GP, and 17.1 % did not seek immediate care at any healthcare provider.

### Engagement in Care

The three most frequent reasons for attending antenatal care were because they felt it was important (70.8 %), because “that’s what you do when you are pregnant” (41.5 %), and because they wanted to know their HIV status (34.9 %) (Table 2).

**Table 2:** Reasons given for attendance and late attendance at ANC and factors that may have facilitated earlier attendance

Reasons attend ANC (n = 198)	(%)	Reasons attend late (n = 106/107)	(%)	Factors that may have enabled earlier attendance			
				Personal factors (n = 102/107)	(%)	Clinic factors (n = 101/107)	(%)
It is important to go to ANC	72.7	Not know pregnant	21.7	No personal factors would have prompted earlier attendance	22.5	Nothing about the clinic would have prompted earlier attendance	58.4
That’s what you do when pregnant	40.9	No time	20.8	Earlier recognition of pregnancy	22.5	More convenient opening and closing times	12.9
To check HIV status	33.3	Attending GP initially	17.0	If not feel well	20.6	Closer proximity to clinic	10.9
Need ANC card to get a bed in labour ward	28.9	Kept putting it off	17.0	If had time to attend	20.6	Friendly clinic staff	7.9
To Prevent HIV infection in baby	15.2	Not know had to go any earlier	7.5	If told or knew had to go sooner	12.7	Shorter waiting time at clinic	6.9
To check personal health/health of baby^a^	14.6	Not know where to go	6.6	If thought there may be problem with baby	7.8	If liked clinic	5.9
Previous obstetric problem^a^	10.1	Not think that it was necessary to go earlier	3.8	Encouragement/support from others	2.9	Money	4.0
Other	8.1	Would be sent away from^a^ clinic if attend any earlier	3.8	If thought it was necessary	2.0	Other	10.9
		Not like the clinic	1.9	Other	15.7		
		Other	14.2				

Frequencies do not add up to 100% as women were allowed to give multiple responses

^a^Responses that were spontaneously raised by women and not an option on the questionnaire

The most frequent reasons given for late attendance were that they did not know they were pregnant (21.7 %), they had no time (20.8 %), and they were seeing a GP for their antenatal care in the first few months (17.0 %). Interestingly, a few women spontaneously mentioned that they had attended late because they knew that they would not be seen at the clinic if they attended any earlier.

Table 2 also shows personal and clinic factors that women felt may have enabled or prompted them to attend ANC earlier. Earlier pregnancy recognition was the most frequently cited personal factor (22.5 %), however the same proportion of women said that no personal factors would have made them attend earlier and an even larger proportion of women (58.4 %) could not think of any clinic based factors that would have encouraged earlier attendance. Only three women mentioned finances as a barrier.

Of the 10 women who did not attend an antenatal clinic, four had received antenatal care from their GP because they either did not like the clinic or were sent away from the clinic. Only one woman said she had no intention of going to antenatal care, two kept putting it off till it was too late, two did not know they were pregnant until quite late and one described herself as being too depressed about the pregnancy to seek care.

### Women’s Experience of Care

Median gestational age at first visit to the clinic was 5 months (IQR 4−6 months), while median gestational age at first booking appointment was 6 months (IQR 4–7 months). A large proportion of women (49.0 %) reported that they were not booked or referred at their first clinic visit. Responses to an open ended question about the reasons for this fell into the following categories: the need to make a booking appointment (55.7 %), lack of an identity document (16.5 %), the clinic operating system e.g. they had reached their limit for the day, they were not accepting new patients or not conducting ANC on that day of the week (14.4 %), and attending “too early” into their pregnancy (8.2 %). Other reasons included clinic workers on strike, and attending a clinic outside of their locality (5.2 %). In addition, 7.1 % reported visiting antenatal clinics between three and six times before they were able to secure a booking.

**Fig. 1:**
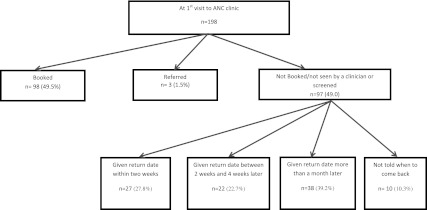
Women’s booking experience at first clinic visit

**Fig. 2:**
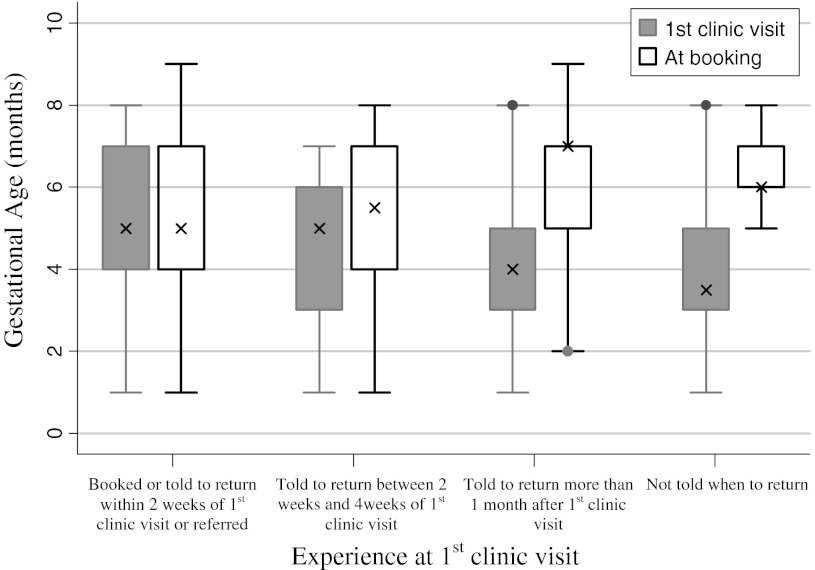
Comparison of gestational age at 1st clinic visit and at booking by women’s experience at first clinic visit. Women who were told to return more than 1 month later or not given return date had 3 month gap between 1st ANC contact and being seen by a health care worker

Of the 97 women not booked or referred at their first visit to the clinic, 39.2 % were told to return more than a month later (Fig. 1). The median gestational age of these women at first clinic visit was 4 months (IQR 3–5 months), while median gestational age at booking was 7 months (IQR 5–7 months) (Fig. 2). Women who were not born in South Africa were also more likely to be given return dates of 2 weeks and greater compared to women for whom South Africa was their country of birth (45.1 % vs. 28.5, χ^2^(1, 198) = 5.84, *p* = 0.02). In addition, of the eight women who were told that they had come too early in their pregnancy to be booked, despite having gestational ages of between 2 and 4 months, four of them reported finally being booked at 7 months and one woman was booked at 9 months.

Women were also asked about a series of checks that should be completed at their booking visit based on the DOH basic antenatal care (BANC) guidelines. At booking, which may or may not be the first contact with the antenatal clinic, 97.0 % reported having a blood test, 97.0 % had blood pressure measurements taken, 96.5 % had their antenatal cards completed, 96.5 % were asked to give a urine sample, 93.4 % had a physical exam, 89.4 % were offered an HIV test and 67.2 % recalled being told about danger signs to be aware of during pregnancy. Only 118 (59.6 %) women recalled having all of the above mentioned routine checks done at booking.

## Discussion

### Health System Operation as a Barrier to Early Initiation

MTCT and HIV related maternal and infant mortality and morbidity may be linked to timing of HIV interventions within pregnancy [19, 20] therefore optimal usage of first contact with health services is imperative if South Africa is to make progress towards *Millennium Development Goals* 4 and 5. It is therefore worth noting that at first contact with clinics, and particularly in a country where it is widely known that women tend to access antenatal care services late [21], many women are being asked to return on another day in order to be booked into the clinic. About half the women reported that no routine checks or screening were conducted at their first visit to the clinic and almost 40 % of those not booked at the first visit were asked to return more than a month later. Of importance is the 3 months difference between median gestational age at first clinic presentation and median gestational age at actual booking for these women. In addition, women whose country of birth was not South Africa were more frequently given return dates >2 weeks after their first visit to the clinic. On examination of the reasons given to women for the delay in provision of care, most are either avoidable or not in line with government antenatal care guidelines. The DOH maternal health guidelines state that antenatal care should begin at a woman’s first visit to the clinic even if the first visit was to confirm a pregnancy [7, 22]. In addition, it is DOH policy that no one should be refused public healthcare with or without a South African identity document.

Furthermore, the operating system, particularly quotas and opening hours/days, in place at some clinics needs to be urgently addressed as this was one of the more frequently cited reasons for not being able to be booked into a clinic as well as for having to make several trips to clinics. As women are encouraged to attend their closest antenatal clinic this may present an additional external barrier to early initiation of antenatal care.

A few women mentioned that they were told they could not be booked into the clinic as they were still “too early” in their pregnancy to be booked, most of these women ended up being booked in their third trimester. Similar clinic based barriers to early initiation have also been noted in other South African studies including inconsistencies in when women were told to book ranging from 3 to 7 months [23, 24], and being asked to return at a later date in order to be booked and clinic quota systems. As noted in the maternal health situation analysis by Penn-Kekana et al. [25], these findings suggest there is a gap between policy development and implementation and therefore the need for stronger communication strategies, and more effective management, in particular, better monitoring and supervision.

### Health Seeking Behaviour as a Barrier to Early Initiation

Antenatal care coverage is known to be high in South Africa [21] and this study confirms this, 97 % of women in our study were seen at least once for ANC either at a public antenatal care clinic or by a private GP. Most women were seen at least four times for ANC at an antenatal clinic as recommended by WHO [8].

A majority of women felt that it was important to attend ANC which is clearly reflected in the high antenatal coverage however, a large proportion sought ANC at an ANC clinic late and many did not feel that there was anything either personal or clinic related that would have encouraged them to attend earlier. This apparent dichotomy between seeing ANC as important and late initiation is one that needs further investigation and could not be fully explored in this study, however it may in part be explained by women’s understanding of the role of ANC. Other studies have shown that women may view early attendance as unnecessary if there are no medical concerns, in other words, more as curative than preventative [13, 24, 26] and may value antenatal care more for the role it plays in ensuring a safe delivery and enabling prompt access to care in labour and delivery [14, 24, 27]. Indeed, almost a third of women who attended ANC said they did so in order to get an antenatal card and therefore expedite their access to care when they went into labour and a fifth said they would have attended earlier if they felt unwell.

One of the more frequently cited reasons for both late attendance and prompts to earlier attendance was a lack of recognition of pregnancy. More than a fifth of women who attended late said they did so because they did not know they were pregnant. The same proportion said they would have attended earlier if they had realised their pregnancy sooner. Lack of pregnancy recognition as a barrier to antenatal care has also been noted by others [14, 24, 28]. The promotion of early recognition of pregnancy is clearly important as it may motivate women to seek care earlier and may also provide the impetus to undertake positive lifestyle changes such as decreasing alcohol consumption or improving their dietary intake which in turn may decrease the risk of adverse pregnancy outcomes [29].

Issues of access such as money and transport did not feature highly as reasons for late or non-attendance for antenatal care in this setting probably because ANC is free in South Africa and this being an urban environment where all primary healthcare centres offer ANC, most women would live close to a clinic. Time does however seem to be a concern and this may be a reflection of the clinic operating system including long waiting times and opening hours as has been observed in other studies [24, 30, 31]. It is understandable that these would be important barriers to early initiation particularly in the context of women being apathetic to antenatal care in the first few months of pregnancy and may also explain why women who were paid employees were least likely to attend early.

Almost 60 % of late attendees said there was nothing they could think of about the clinic system that would make them attend ANC earlier. Additionally, although a large proportion of women were unable to be booked at their first visit to the clinic, none of them mentioned this as a barrier to earlier antenatal care. These findings suggest that women expect or even accept this as a norm and further investigation into understanding this is warranted.

Both the high ANC coverage and the expression that most women value the need to attend ANC may be attributed to the government’s strong drive to promote maternal health. Nevertheless, there is still clearly a continuing need for increased community engagement as evidenced by the large number of women being unaware of their pregnancy until late, the high levels of unplanned pregnancies, and the high proportion of women who said they went for antenatal care in order to find out their HIV status. In addition, women should know what health care to expect and where this is not provided she should be empowered sufficiently to demand appropriate care.

## Limitations

Firstly, this study is based on self-reports and asks about past history, without verification of clinical records, and may therefore be subject to recall bias, however as women were interviewed in the immediate post-partum period and pregnancy being an important time in most women’s lives, we expect our results to be as close as possible to a true reflection of women’s experiences. Secondly, this is a descriptive cross-sectional study and as such we could only assess associations and causality cannot be inferred. Lastly, as women were interviewed after childbirth, we were unable to get the views of women who died antenatally or during childbirth. Nevertheless, as most women attend ANC, and most deaths occur postnatally, we do not expect this to have had a significant effect on our results and believe our study is generalisable to women delivering in public labour wards in urban South Africa.

## Conclusions

Antenatal care attendance in the inner-city of Johannesburg is high and most women are seen at least four times while pregnant, however, a large proportion of women still attend late. As has been noted in other studies, this may in part be attributed to women’s perceptions of the role of antenatal care however, clinic booking procedures appear to compound the situation. In a country where a third of pregnant women are HIV infected, early ANC is vital in order to optimise ART initiation and thereby reduce maternal mortality and paediatric HIV infection. Implementation of existing policies needs to be reinforced, clinics need to take into account the demands on women’s time by improving operating procedures and women need to be empowered to demand better services.

## References

[1] WHO Global Health Observatory [Internet]. Available from: http://www.who.int/gho/countries/zaf/country_profiles/en/index.html. Accessed 27 July 2011.

[2] World Bank data bank [Internet]. Available from: http://data.worldbank.org/indicator/SH.STA.MMRT/countries/1W-XT-ZA?display=graph. Accessed 27 July 2011.

[3] National Department of Health (2007). Saving mothers: Third report on confidential inquiries into maternal deaths in South Africa, 2002–04.

[4] WHO and UNICEF. Antenatal care in developing countries: Promises, achievements and missed opportunities. *WHO/UNICEF 2003.* Available from: http://www.who.int/reproductive_health/global_monitoring/data.html. Accessed 27 July 2011.

[5] Villar J, Bergsjo P (1997). Scientific basis for the content of routine antenatal care. I. Philosophy, recent studies, and power to eliminate or alleviate adverse maternal outcomes. Acta Obstetricia et Gynecologica Scandinavica.

[6] Pattinson, R. C. (2009). *Saving babies 2006–2007: Sixth perinatal care survey of South Africa*. Pretoria: Tshepesa Press.

[7] Pattinson RC (2007). Basic antenatal care handbook.

[8] Villar, J., Bergsjo, P. (2002). WHO antenatal care randomised trial: Manual for the implementation of the new model. WHO programme to map best reproductive health practices.

[9] Black V, Brooke S, Chersich MF (2009). Effect of human immunodeficiency virus treatment on maternal mortality at a tertiary center in South Africa: A 5-year audit. Obstetrics and Gynecology.

[10] National Department of Health (2011). The national antenatal sentinel HIV and syphilis prevalence survey, South Africa, 2010.

[11] Townsend CL, Cortina-Borja M, Peckham CS, de Ruiter A, Lyall H, Tookey PA (2008). Low rates of mother-to-child transmission of HIV following effective pregnancy interventions in the United Kingdom and Ireland, 2000–2006. AIDS.

[12] Hoffman RM, Black V, Technau K, van der Merwe KJ, Currier J, Coovadia A, Chersich M (2010). Effects of highly active antiretroviral therapy duration and regimen on risk for mother-to-child transmission of HIV in Johannesburg, South Africa. Journal of Acquired Immune Deficiency Syndromes.

[13] Fitzgerald FC, Bekker L-G, Kaplan R, Myer L, Lawn SD, Wood R (2010). Mother-to-child transmission of HIV in a community-based antiretroviral clinic in South Africa. South African Medical Journal.

[14] Myer L, Harrison A (2003). Why do women seek antenatal care late? Perspectives from rural South Africa. Journal of Midwifery and Women’s Health.

[15] Sibeko S, Moodley J (2006). Healthcare attendance patterns by pregnant women in Durban, South Africa. South African Family Practice.

[16] Ndidi EP, Oseremen IG (2010). Reasons given by pregnant women for late initiation of antenatal care in the Niger delta, Nigeria. Ghana Medical Journal.

[17] Ochako R, Fotso J, Ikamari L, Khasakhala A (2011). Utilization of maternal health services among young women in Kenya: Insights from the Kenya Demographic and Health Survey, 2003. BMC Pregnancy and Childbirth.

[18] Adamu YM, Salihu HM (2002). Barriers to the use of antenatal and obstetric care services in rural Kano, Nigeria. Journal of Obstetrics and Gynaecology.

[19] Marazzi MC, Palombi L, Nielsen-Saines K, Haswell J, Zimba I, Abdul Magid N (2011). Extended antenatal use of triple antiretroviral therapy for prevention of HIV-1 mother-to-child-transmission correlates with favourable pregnancy outcomes. AIDS.

[20] Chibwesha C, Giganti M, Putta N, Chintu N, Mulindwa J, Dorton B (2011). Optimal time on HAART for prevention of mother-to-child transmission of HIV. Journal of Acquired Immune Deficiency Syndromes.

[21] Department of Health, Medical Research Council, OrcMacro. (2007). *South Africa Demographic and Health Survey 2003.* Pretoria: Department of Health.

[22] Gauteng Department of Health, Directorate: mother and child health, *Antenatal care policy document.* Accessed from University of Pretoria website: http://www.ais.up.ac.za/med/block9/antenatalcarepolicy.pdf. Accessed 23 May 2011.

[23] Abrahams N, Jewkes R, Mvo Z (2001). Health care—seeking practices of pregnant women and the role of the midwife in Cape Town, South Africa. The Journal of Midwifery and Women’s Health.

[24] Gatsinzi S, Maharaj P (2008). Women’s experiences of maternal and child health and family planning services in KwaZulu-Natal. Curationis.

[25] Penn-Kekana L, McPake B, Parkhurst J (2007). Improving maternal health: Getting what works to happen. Reproductive Health Matters.

[26] Waiswa P, Kemigisa M, Kiguli J, Naikoba S, Pariyo GW, Peterson S (2008). Acceptability of evidence-based neonatal care practices in rural Uganda—implications for programming. BMC Pregnancy and Childbirth.

[27] Mrisho M, Obrist B, Armstrong Schellenberg J, Haws RA, Mushi AK, Mshinda H (2009). The use of antenatal and postnatal care: perspectives and experiences of women and health care providers in rural southern Tanzania. BMC Pregnancy and Childbirth.

[28] Downe S, Finlayson K, Walsh D, Lavender T (2009). ‘Weighing up and balancing out’: A meta-synthesis of barriers to antenatal care for marginalised women in high-income countries. BJOG.

[29] Ayoola, A. B, Stommel, M., Nettleman, M. D. (2009). Late recognition of pregnancy as a predictor of adverse birth outcomes. *American journal of**obstetrics and gynecology*, *201,* 156.e1−156.e6. 10.1016/j.ajog.2009.05.011 19646566

[30] Tlebere P, Jackson D, Loveday M, Matizirofa L, Mbombo N, Doherty T (2007). Community-based situation analysis of maternal and neonatal care in South Africa to explore factors that impact utilization of maternal health services. The Journal of Midwifery and Women’s Health.

[31] Jewkes R, Abrahams N, Mvo Z (1998). Why do nurses abuse patients? Reflections from South African obstetric services. Social Science and Medicine.

